# Biological and biomimetic surfaces: adhesion, friction and wetting phenomena

**DOI:** 10.3762/bjnano.10.48

**Published:** 2019-02-15

**Authors:** Stanislav N Gorb, Kerstin Koch, Lars Heepe

**Affiliations:** 1 Department of Functional Morphology and Biomechanics, Zoological Institute of the University of Kiel, Am Botanischen Garten 9, 24118 Kiel, Germany; 2 Rhine-Waal-University of Applied Sciences, Marie Currie Str. 1, 47533 Kleve, Germany

**Keywords:** adhesion, air retention, contact mechanics, fluid transport, friction, functional gradients, wetting

This Thematic Series is the continuation of the previous series on the broad topic of biological and bioinspired materials and surfaces [1–3]. This collection of articles displays a current cross section of recent developments in this highly diverse and interdisciplinary field of research.

The articles highlight recent achievements in the understanding of animal and plant surfaces in the broadest context of adhesion, friction, and wetting phenomena on one hand. On the other hand, they document the recent progress of transferring these findings into artificial materials and surfaces using advanced fabrication methods and highlight the broad spectrum of both experimental and theoretical techniques to characterize biological and bioinspired surfaces.

Out of the 19 collected articles, eight are devoted to surface-related effects in animal and plant surfaces, such as sandfish scales, wings of a ladybird beetle, tarsi of burying beetles, attachment devices of a sea star and a sea urchin, elytra of a backswimmer, leaves of an ice plant, and the wax layer of sacred lotus leaves.

Seven of the collected articles are devoted to surface-related effects in engineered surfaces, such as multilayered composites, carbon nanofibers, textured steel surfaces, and micropatterned elastomer surfaces.

Three articles present recent work on the development of a novel fabrication technique for biomaterials and of novel flow and pressure sensors.

While most of the articles represent experimental work, two are devoted to theoretical and numerical work on the adhesion of rough brush systems and the friction of functionally graded materials.

The metrics mentioned above illustrate that this compilation of articles reflects the high diversity and interdisciplinary nature of this field of research, as it combines approaches from biology, physics, engineering, tribology and materials science. The articles of this Thematic Series are intended to be of interest to both engineers and physicists who work with biological systems as well as to biologists who study the physics of friction and adhesion.

We would like to thank all the authors for contributing their beautiful work to this Thematic Series! Moreover, we are grateful to all referees for their promptly provided reports that facilitated the high quality of the manuscripts and allowed us to publish these works in a timely manner. Finally, we thank the Editorial Office at the Beilstein-Institut for their continuous great support.

Stanislav N. Gorb, Kerstin Koch, and Lars Heepe

Kiel and Kleve, December 2018
